# Supplementary material for: Sequence–Activity Relationship of ATCUN Peptides in the Context of Alzheimer’s Disease
Source: Molecules. 2022 Nov 15;27(22):7903. doi: 10.3390/molecules27227903 (PMC9698028; doi:10.3390/molecules27227903)
Supplement: Supplementary file 1 [file molecules-27-07903-s001.zip › molecules-2000245-supplementary.pdf]

Supporting Information

Sequence–Activity Relationship of ATCUN Peptides in the Context of Alzheimer's Disease

Margot Lefèvre, Kyangwi P. Malikidogo, Charlène Esmieu and Christelle Hureau *

CNRS, LCC (Laboratoire de Chimie de Coordination), 205 Route de Narbonne, BP 44099
31077 Toulouse Cedex 4, France
* Correspondence: christelle.hureau@lcc-toulouse.fr.

ATCUN peptide characterizations.

GVHW-NH_2 :

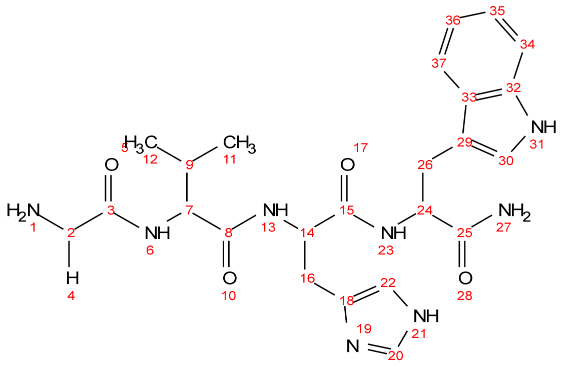

^{1}H-NMR (D_2O, 400 MHz): δ (ppm) 8.55 (d, $^{4}J_{20\text{-}22}$=1.4 Hz, 1H), 7.67 (d, $^{3}J_{34\text{-}35}$=7.8, $^{4}J_{34\text{-}36}$=1.0 Hz, 1H), 7.51 (d, $^{3}J_{37\text{-}36}$=8.1, $^{4}J_{37\text{-}35}$=0.9 Hz, 1H), 7.30 – 7.20 (m, 3H), 7.17 (td, $^{3}J_{35\text{-}36}$=8.0, $^{3}J_{35\text{-}34}$=7.0, $^{4}J_{34\text{-}36}$=1.1 Hz, 1H), 4.73 – 4.62 (m, 2H), 4.03 (d, $^{3}J_{7\text{-}9}$=7.1 Hz, 1H), 3.92 – 3.78 (m, 2H), 3.36 – 3.03 (m, 4H), 1.85 (hept, $^{3}J_{9\text{-}11,12}$=6.8 Hz, 1H), 0.79 (d, $^{3}J_{11,12\text{-}9}$=6.8 Hz, 3H), 0.70 (d, $^{3}J_{11,12\text{-}9}$=6.8 Hz, 3H).

^{13}C-NMR (D_2O, 101 MHz): δ (ppm) 176.87, 174.22, 170.57, 162.19, 136.44, 133.42, 129.01, 126.52, 124.48, 122.68, 119.29, 118.23, 116.32, 111.96, 108.66, 59.52, 54.82, 52.18, 41.88, 28.27, 27.35.

(+) ESI-MS calculated for $C_{24}H_{32}N_8O_4$: $[M + H]^+$ m/z 497.25, Experimental $[M + H]^+$ m/z 497.3.

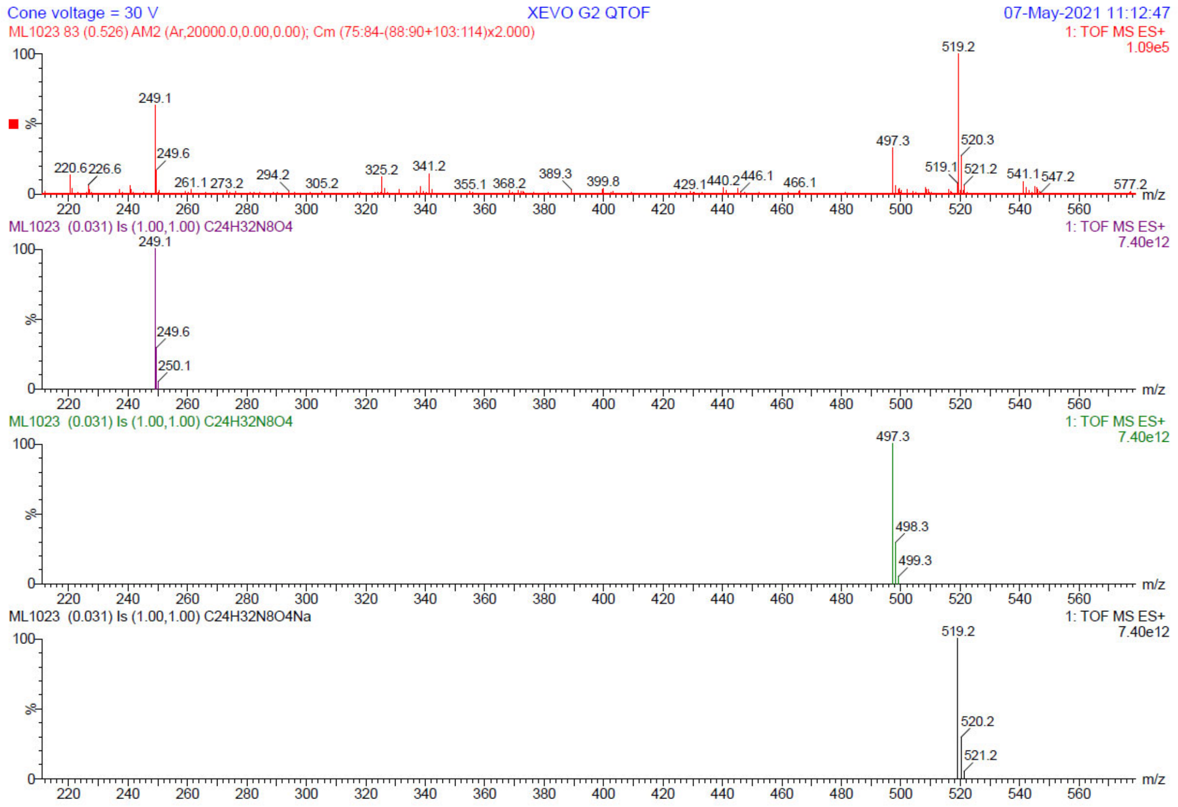

VGHW-NH$_2$:

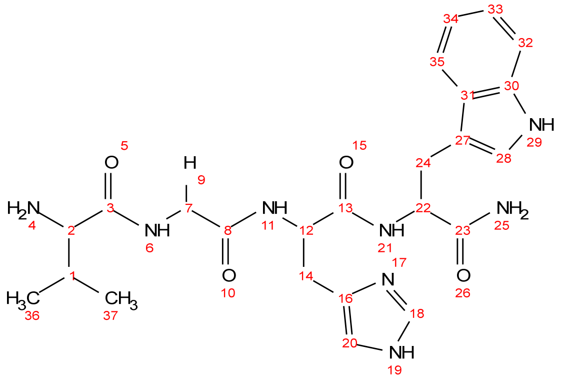

^{1}H-NMR (D_2O, 400 MHz): δ (ppm) 8.49 (d, $^{4}J_{18\text{-}20}$=1.5 Hz, 1H), 7.67 (d, $^{3}J_{32\text{-}33}$=7.9, $^{3}J_{32\text{-}34}$=1.1 Hz, 1H), 7.51 (d, $^{3}J_{35\text{-}34}$=8.2, $^{3}J_{35\text{-}33}$ = 0.9 Hz, 1H), 7.29 – 7.22 (m, 2H), 7.16 (td, $^{3}J_{33\text{-}34}$=8.0, $^{3}J_{33\text{-}32}$7.0, 1.1 Hz, 1H), 7.07 (d, $^{3}J_{20\text{-}18}$=1.4 Hz, 1H), 4.67 (td, $^{3}J_{22\text{-}24}$=8.4, $^{3}J_{22\text{-}24}$=6.2 Hz, 1H), 4.60 (td, $^{3}J_{12\text{-}14}$=7.7, $^{3}J_{12\text{-}14}$=6.7 Hz, 1H), 3.97 – 3.78 (m, 3H), 3.37 – 3.15 (m, 1H), 3.13 – 2.91 (m, 1H), 2.22 (hept, 1H), 1.03 (dd, $^{3}J_{36,37\text{-}1}$=6.9, $^{4}J_{36\text{-}37}$=1.6 Hz, 6H)

^{13}C-NMR (D_2O, 101 MHz): δ (ppm) 175.75, 171.08, 170.42, 169.84, 136.07, 133.34, 128.01, 126.85, 124.53, 121.93, 119.35, 118.33, 117.13, 111.86, 108.97, 58.52, 54.06, 52.54, 42.04, 29.87, 27.05, 26.27, 17.47, 16.86.

(+) ESI-MS calculated for $C_{24}H_{32}N_8O_4$: $[M + H]^+$ m/z 497.25, Experimental $[M + H]^+$ m/z 497.3.

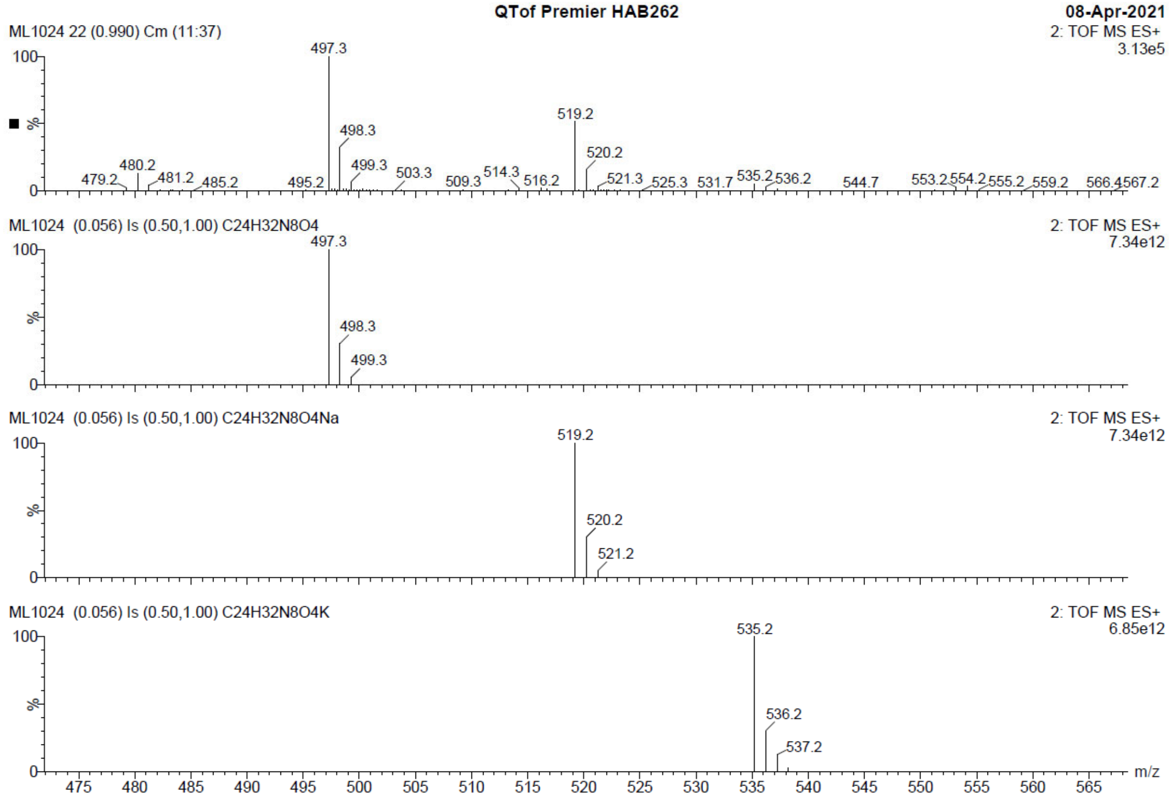

GGHW-NH$_2$:

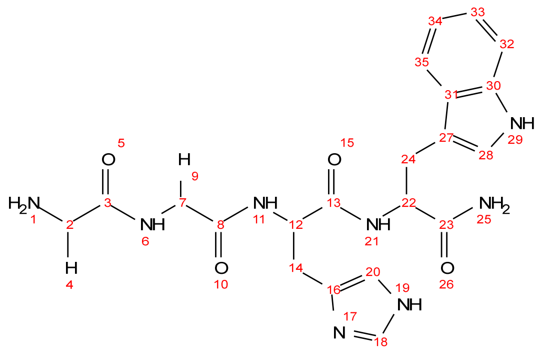

^{1}H-NMR (D_2O, 400 MHz): δ (ppm) 8.39 (d, $^4J_{18\text{-}20}$ = 2.0 Hz, 1H), 7.68 (dt, $^3J_{32\text{-}33}$ =7.9, $^4J_{32\text{-}34}$ = 1.1 Hz, 1H), 7.52 (dt, $^3J_{35\text{-}34}$ =8.2, $^4J_{35\text{-}33}$ = 0.9 Hz, 1H), 7.31 – 7.21 (m, 2H), 7.18 (td, $^3J_{34\text{-}33}$ =8.0, $^3J_{34\text{-}35}$ = 7.0, $^4J_{34\text{-}32}$ = 1.1 Hz, 2H), 7.07 – 6.93 (m, 1H), 4.68 (td, $^3J_{12\text{-}14}$ =8.3, $^3J_{12\text{-}14}$ =6.3 Hz, 1H), 4.61 (td, 1H), 3.98 – 3.78 (m, 4H), 3.39 – 3.16 (m, 2H), 3.11 – 2.96 (m, 2H)

^{13}C-NMR (D_2O, 101 MHz): δ (ppm) 175.67, 173.02, 170.87, 167.09, 136.14, 133.42, 128.03, 126.72, 124.44, 121.93, 119.29, 118.23, 117.23, 111.90, 108.66, 59.42, 53.92, 52.18, 40.18, 30.10, 27.17, 26.25, 18.01, 17.31.

(+) ESI-MS calculated for $C_{21}H_{26}N_8O_4$: $[M + H]^+$ m/z 455.21, Experimental $[M + H]^+$ m/z 455.3.

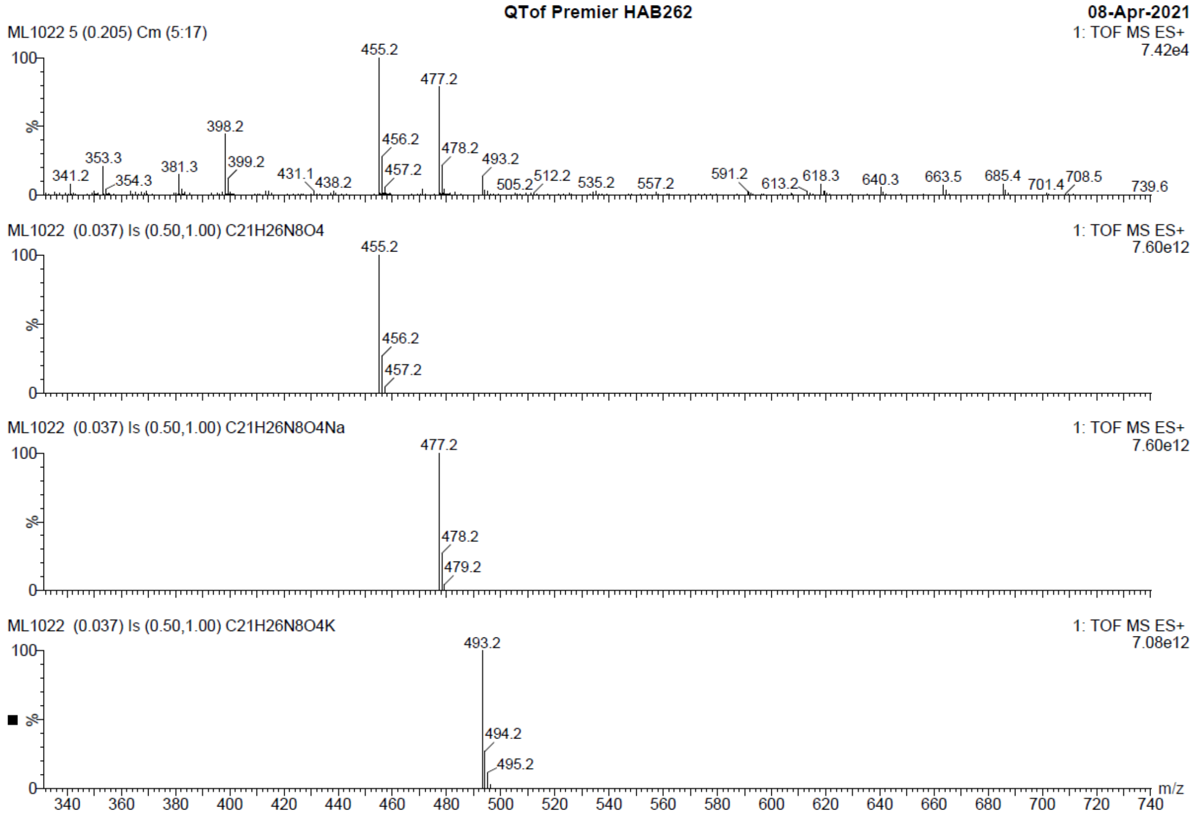

DAHW-NH$_2$:

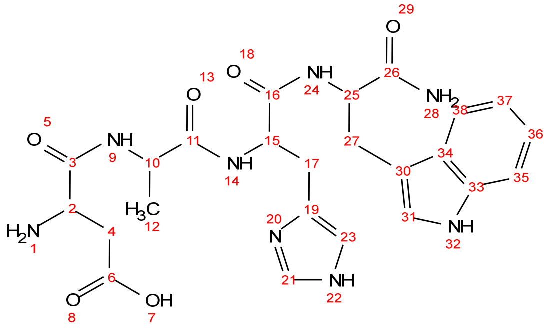

^{1}H-NMR (D_2O, 400 MHz): δ (ppm) 8.50 (d, $^4J_{21-23}$=1.3 Hz, 1H), 7.64 (td, $^3J_{35-36}$=7.9, $^4J_{35-37}$=1.1 Hz, 1H), 7.47 (td, $^3J_{38-37}$=8.2, $^4J_{38-36}$=1.1 Hz, 1H), 7.26 – 7.18 (m, 2H), 7.18 – 7.07 (m, 2H), 4.65 (td, $^3J_{27-25}$ =7.7, $^3J_{27-25}$=6.4 Hz, 1H), 4.58 (td, $^3J_{15-17}$=8.0, $^3J_{15-17}$=6.7 Hz, 1H), 4.30 (td, $^3J_{2-4}$=7.5, $^3J_{2-4}$=5.2 Hz, 1H), 4.23 (q, $^3J_{10-12}$=7.2 Hz, 1H), 3.35 – 2.87 (m, 6H), 1.21 (d, $^3J_{12-10}$ =7.3 Hz, 3H)

^{13}C-NMR (D_2O, 101 MHz): δ (ppm) 175.69, 174.03, 172.45, 170.97, 168.22, 136.08, 133.37, 128.03, 126.78, 124.42, 121.93, 119.30, 118.29, 117.19, 111.86, 108.76, 53.90, 52.35, 49.70, 49.21, 34.59, 27.11, 26.21, 16.26

(+) ESI-MS calculated for $C_{24}H_{30}N_8O_6$: $[M + H]^+$ m/z 527,23, Experimental $[M + H]^+$ m/z 527.2.

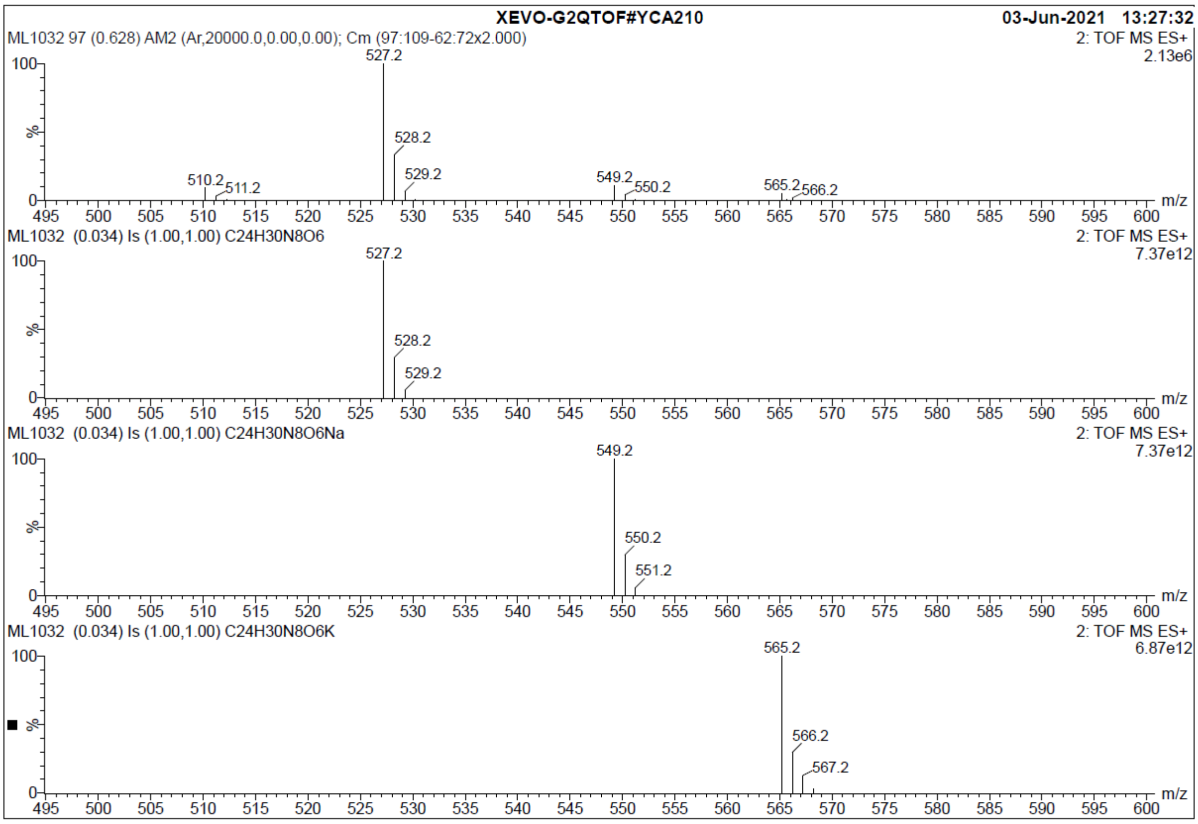

GHHW-NH_2:

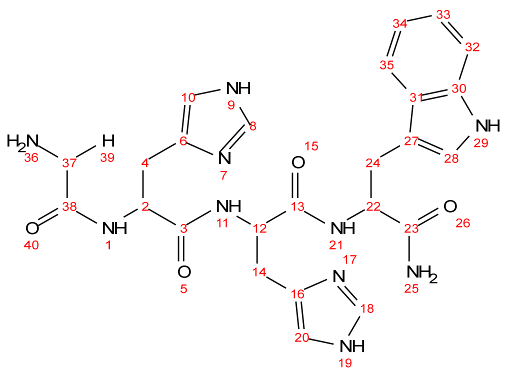

^{1}H-NMR (D_2O, 400 MHz): δ (ppm) 8.41 (d, 1H), 8.29 (d, $^4J_{8\text{-}10}$=1.0 Hz, 1H), 7.59 (d, 1H), 7.32 (d, 1H), 7.18 (s, 1H), 7.14 – 7.00 (m, 3H), 6.76 (d, $^4J_{10\text{-}8}$=1.4 Hz, 1H), 4.62 – 4.53 (m, 2H), 4.46 (t, $^3J_{22\text{-}24}$=7.2 Hz, 1H), 3.77 – 3.64 (m, 3H), 3.28 – 2.70 (m, 6H)

^{13}C-NMR (D_2O, 101 MHz): δ (ppm) 175.71, 171.23, 170.63, 168.69, 136.01, 134.36, 133.43, 127.89, 126.84, 125.27, 124.30, 121.90, 119.30, 118.71, 118.27, 117.15, 111.73, 109.00, 53.84, 52.87, 51.81, 41.90, 27.10, 26.16 – 25.87 (m).

(+) ESI-MS calculated for $C_{25}H_{30}N_{10}O_4$: $[M + H]^+$ m/z 535.25, Experimental $[M + H]^+$ m/z 535.3.

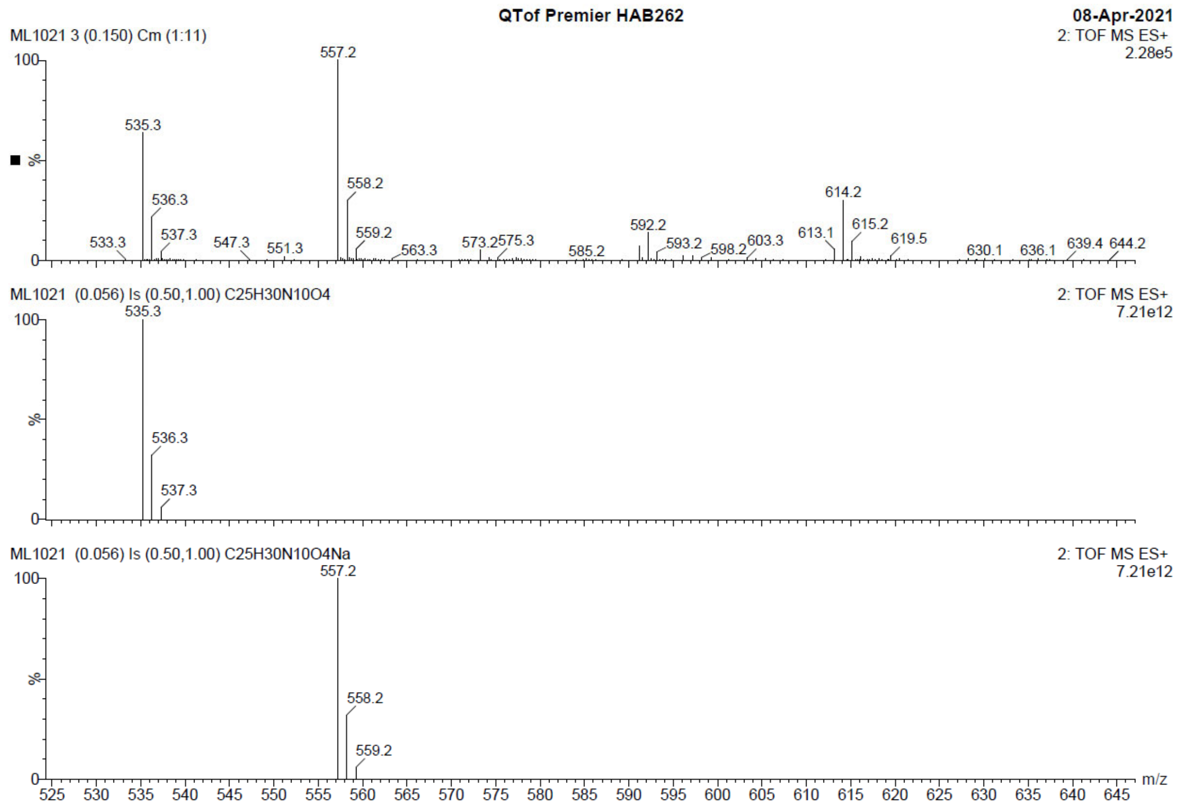

HGHW-NH$_2$:

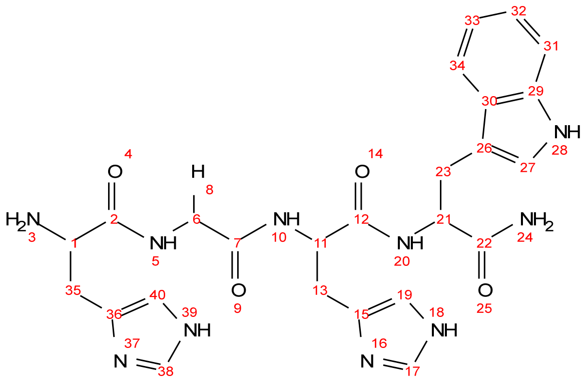

^{1}H-NMR(D_2O, 400 MHz): δ (ppm) 8.55 (d, $^{4}J_{38-40}$ =1.4 Hz, 1H), 8.51 (d, $^{4}J_{17-19}$=1.5 Hz, 1H), 7.65 (d, $^{3}J_{31-32}$=7.9, $^{4}J_{31-33}$ =1.1 Hz, 1H), 7.45 (d, $^{3}J_{34-32}$=8.2, $^{4}J_{34-32}$ =1.0 Hz, 1H), 7.41 (d, $^{4}J_{40-38}$ =1.4 Hz, 1H), 7.26 – 7.17 (m, 2H), 7.13 (td, $^{3}J_{32-33}$=8.0, $^{3}J_{32-31}$ =7.0, $^{4}J_{32-34}$ =1.1 Hz, 1H), 7.10 (d, $^{4}J_{19-17}$ =1.4 Hz, 1H), 4.71 (td, $^{3}J_{21-23}$ =8.5, $^{3}J_{21-23}$ =6.0 Hz, 1H), 4.59 (t, $^{3}J_{11-13}$ =7.3 Hz, 1H), 4.38 (t, $^{3}J_{1-35}$=6.3 Hz, 1H), 4.09 – 3.74 (m, 2H), 3.41 (d, $^{3}J_{35-1}$ =6.4 Hz, 2H), 3.26 (dd, $^{4}J_{23-27}$ =14.8, $^{3}J_{23-21}$ =6.6 Hz, 2H), 3.08 (d, $^{3}J_{13-11}$=7.3 Hz, 2H)

^{13}C- NMR (D_2O, 101 MHz): δ (ppm) 175.71, 171.23, 170.63, 168.69, 136.01, 134.36, 133.43, 127.89, 126.84, 125.27, 124.30, 121.90, 119.30, 118.71, 118.27, 117.15, 111.73, 109.00, 53.84, 52.87, 51.81, 41.90, 27.10, 26.16 – 25.87 (m).

(+) ESI-MS calculated for $C_{25}H_{30}N_{10}O_4$: $[M + H]^+$ m/z 535.25, Experimental $[M + H]^+$ m/z 535.3.

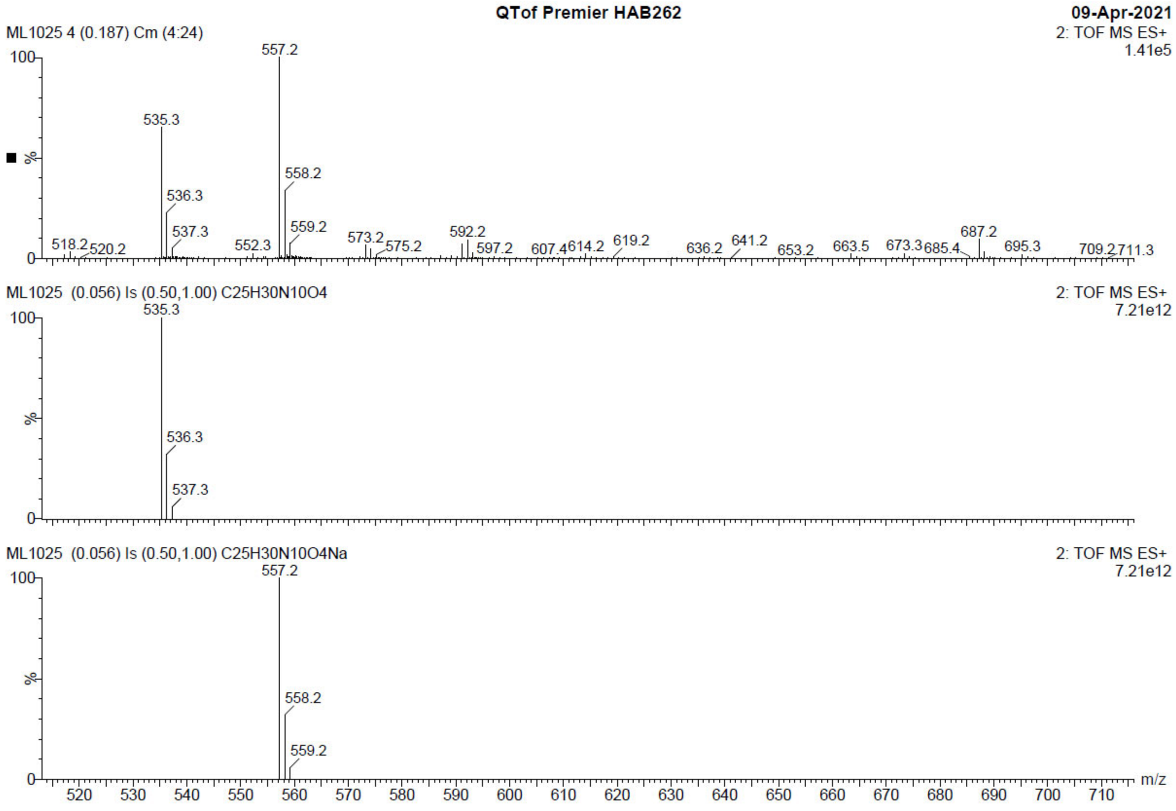

WHHG-NH_2 :

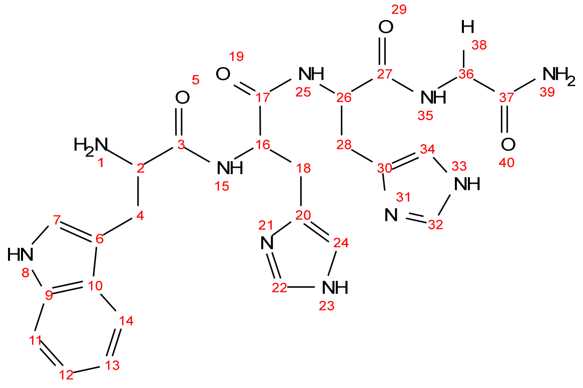

^{1}H-NMR (D_2O, 400 MHz): δ (ppm) 8.42 (d, $^4J_{32\text{-}34}$ =1.0 Hz, 1H), 8.32 (d, $^4J_{22\text{-}24}$=1.0 Hz, 1H), 7.59 (d, 1H), 7.32 (d, 1H), 7.18 (s, 1H), 7.14 – 7.00 (m, 3H), 6.76 (d, $^4J_{24\text{-}22}$=1.2 Hz, 1H), 4.62 – 4.53 (m, 2H), 4.46 (t, $^3J_{2\text{-}4}$=7.2 Hz, 1H), 3.77 – 3.64 (m, 3H), 3.4 – 2.0 (m, 6H)

^{13}C-NMR (D_2O, 101 MHz): δ (ppm) 177.35 174.30, 170.63, 168.69, 136.01, 134.36, 133.43, 127.89, 126.40, 125.27, 123.30, 122.15, 119.30, 118.77, 118.67, 117.15, 111.73, 105.00, 54.98, 52.19, 51.81, 41.90, 27.10, 27.10 – 25.90 (m)

(+) ESI-MS calculated for $C_{25}H_{30}N_{10}O_4$: $[M + H]^+$ m/z 535.25, Experimental $[M + H]^+$ m/z 535.3.

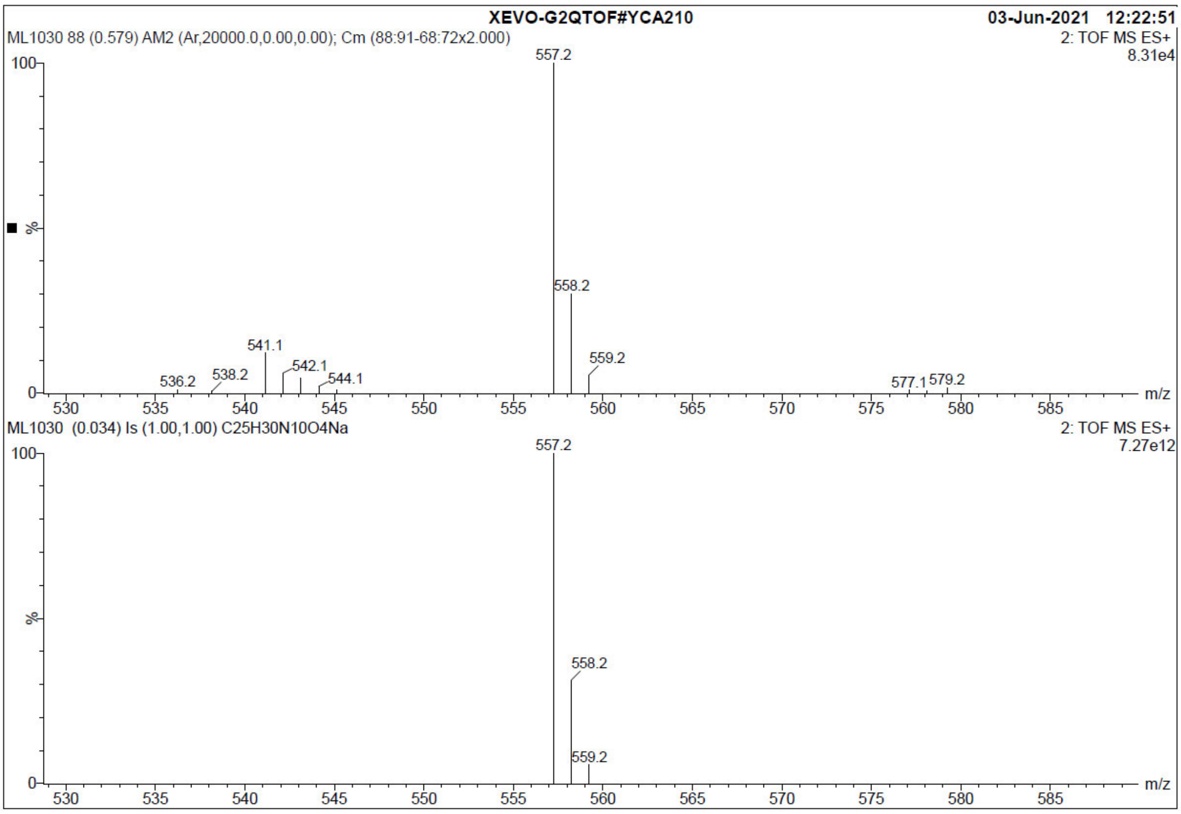

HWHG-NH$_2$:

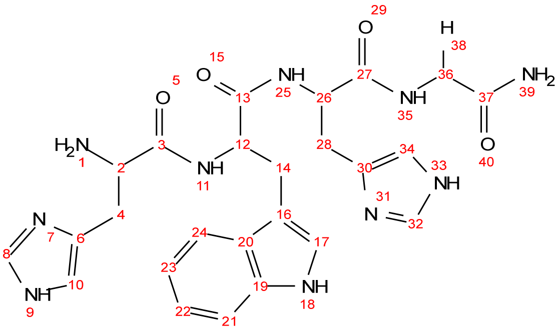

^{1}H-NMR (D_2O, 400 MHz): δ (ppm) 8.65 (d, $^{4}J_{8\text{-}10}$=1.4 Hz, 1H), 8.50 (d, $^{4}J_{32\text{-}24}$=1.4 Hz, 1H), 7.54 (d, $^{3}J_{21\text{-}22}$=8.0, $^{4}J_{21\text{-}23}$=1.2 Hz, 1H), 7.44 (d, $^{3}J_{24\text{-}23}$=8.2 Hz, 1H), 7.39 (d, $^{4}J_{10\text{-}8}$=1.4 Hz, 1H), 7.22 (s, 1H), 7.20 (td, $^{3}J_{23\text{-}24}$=8.2, $^{3}J_{23\text{-}22}$=7.2, $^{4}J_{23\text{-}21}$=1.2 Hz, 1H), 7.17 – 7.07 (m, 2H), 4.60 (td, $^{3}J_{12\text{-}14}$=9.8, $^{3}J_{12\text{-}14}$=6.0 Hz, 1H), 4.44 (td, $^{3}J_{26\text{-}28}$=7.1, $^{3}J_{26\text{-}28}$=6.0 Hz, 1H), 4.32 (t, $^{3}J_{2\text{-}4}$=6.8 Hz, 1H), 3.60 (s, 2H), 3.35 (d, $^{3}J_{4\text{-}2}$=6.9 Hz, 2H), 3.31 – 3.11 (m, 2H), 3.14 – 2.92 (m, 2H)

^{13}C-NMR (D_2O, 101 MHz): δ (ppm) 173.39, 173.08, 170.82, 167.77, 135.88, 134.31, 133.21, 127.97, 126.57, 125.64, 124.61, 122.11, 119.55, 118.50, 118.00, 117.11, 111.86, 107.98, 55.35, 52.24, 51.73, 41.84, 26.72, 26.28 – 25.95 (m)

(+) ESI-MS calculated for $C_{25}H_{30}N_{10}O_4$: $[M + H]^+$ m/z 535.25, Experimental $[M + H]^+$ m/z 535.3.

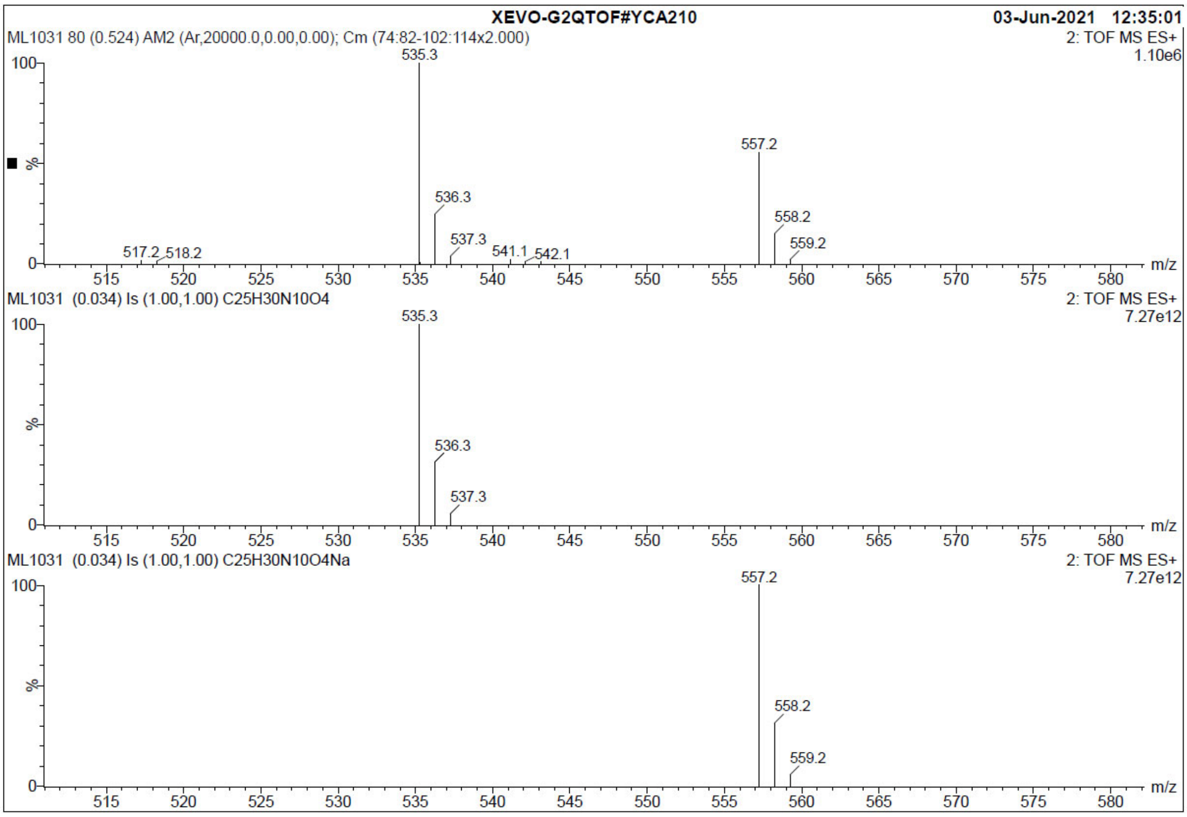

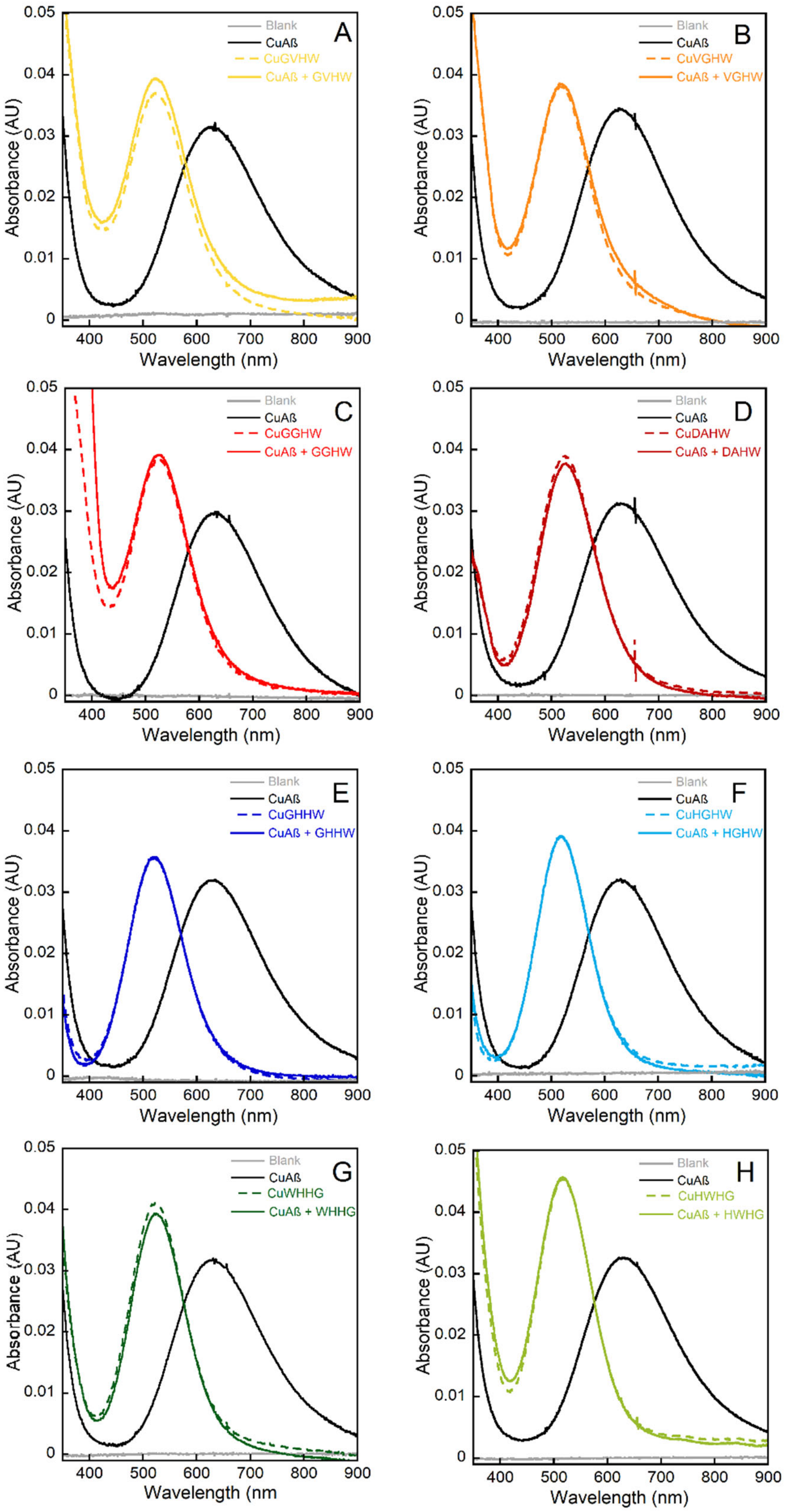

Figure S1. Cu^{II} removal from Cu^{II}(Aβ) by P followed by UV-Vis. Each panel contains spectra of Cu^{II}(Aβ) (black curves), Cu^{II}(P) (GVHW (dashed yellow curves, A), VGHW (dashed orange curves, B), GGHW (dashed red curves, C), DAHW (dashed dark red curves, D), GHHW (dashed dark blue curves, E), HGHW (dashed light blue curves, F), WHHG (dashed dark green curves, G), HWHG (dashed light green curves, H)), and of the addition of P to Cu^{II}(Aβ) with mixing for 5 minutes (thick curves). Experimental conditions: [Cu^{II}] = 400 μM, [P] = [Aβ] = 400 μM, [HEPES] = 100 mM, pH 7.4, T = 25 °C.

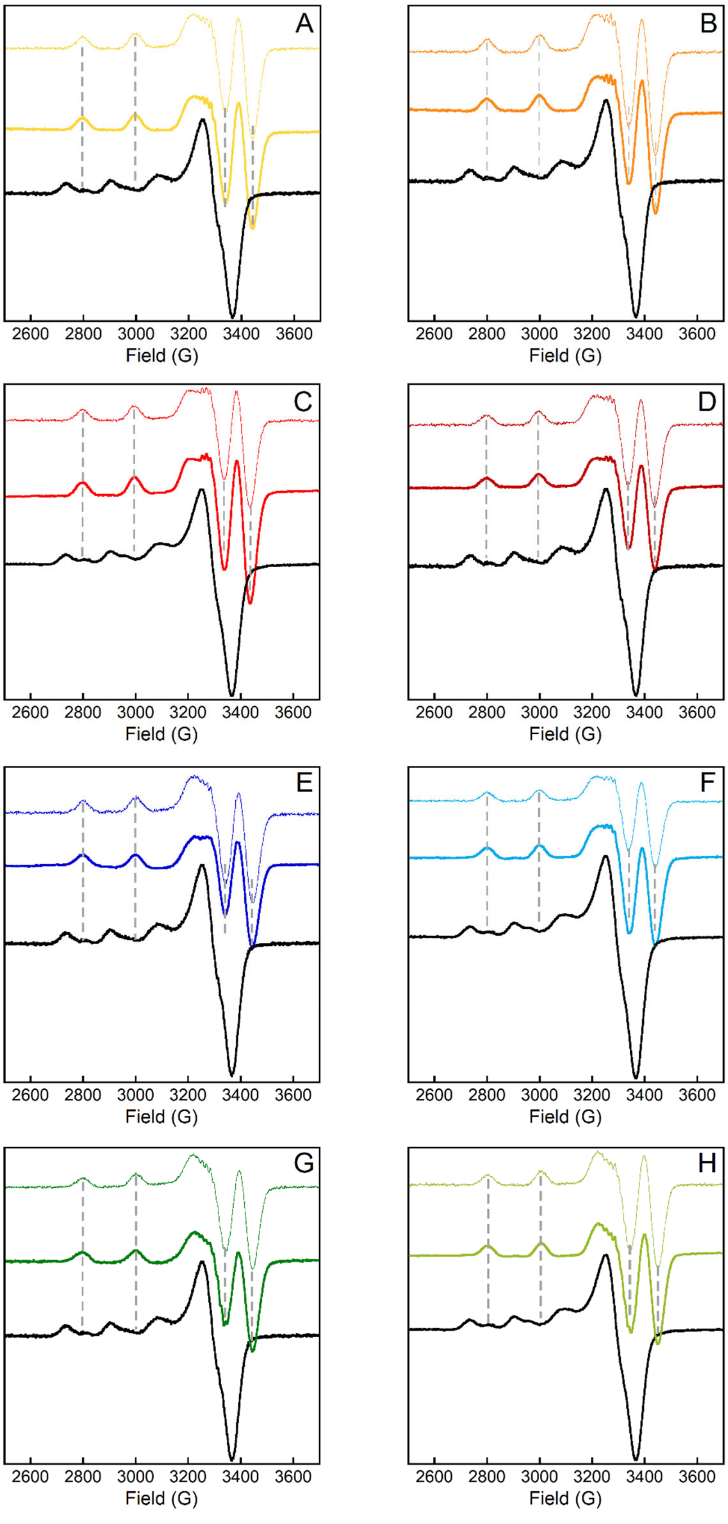

Figure S2. Cu^{II} removal from Cu^{II}(Aβ) by P followed by X-band EPR. Each panel contains spectra of Cu^{II}(Aβ) (black curves), Cu^{II}(P) (GVHW (yellow curves, A), VGHW (orange curves, B), GGHW (red curves, C), DAHW (dark red curves, D), GHHW (dark blue curves, E), HGHW (light blue curves, F), WHHG (dark green curves, G), HWHG (light green curves, H)), and of the addition of P to Cu^{II}(Aβ) and mixing for 5 minutes (thick curve). Experimental conditions: [$^{65}Cu^{II}$] = 500 µM, [P, Aβ] = 600 µM, [HEPES] = 50 mM, pH 7.4, 10 % of glycerol as cryoprotectant, T = 120 K, ν ≈ 9.5 GHz, mod. ampl. = 5 G, microwave power: 5 mW.

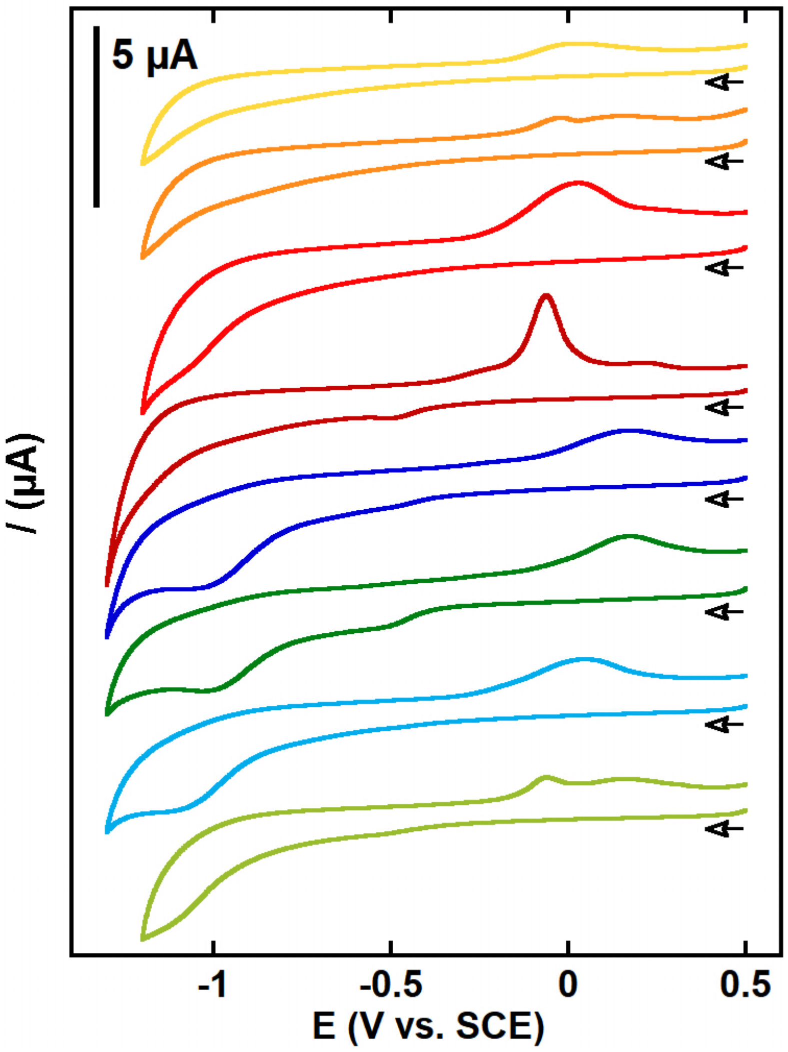

Figure S3. Cyclic voltammograms of Cu(P) (GVHW (yellow curve), VGHW (orange curve), GGHW (red curve), DAHW (dark red curve), GHHW (dark blue curve), HGHW (light blue curve), WHHG (dark green curve), HWHG (light green curve)). The arrows indicate the scanning starting point going first in reduction. [P] = 200 µM, [Cu^{II}] = 180 µM, [HEPES] = 50 mM, pH 7.4, under argon. Scan rate = 100 $mV.s^{-1}$; WE = Glassy carbon, Ref = SCE, CE = Pt wire. First scans are shown.

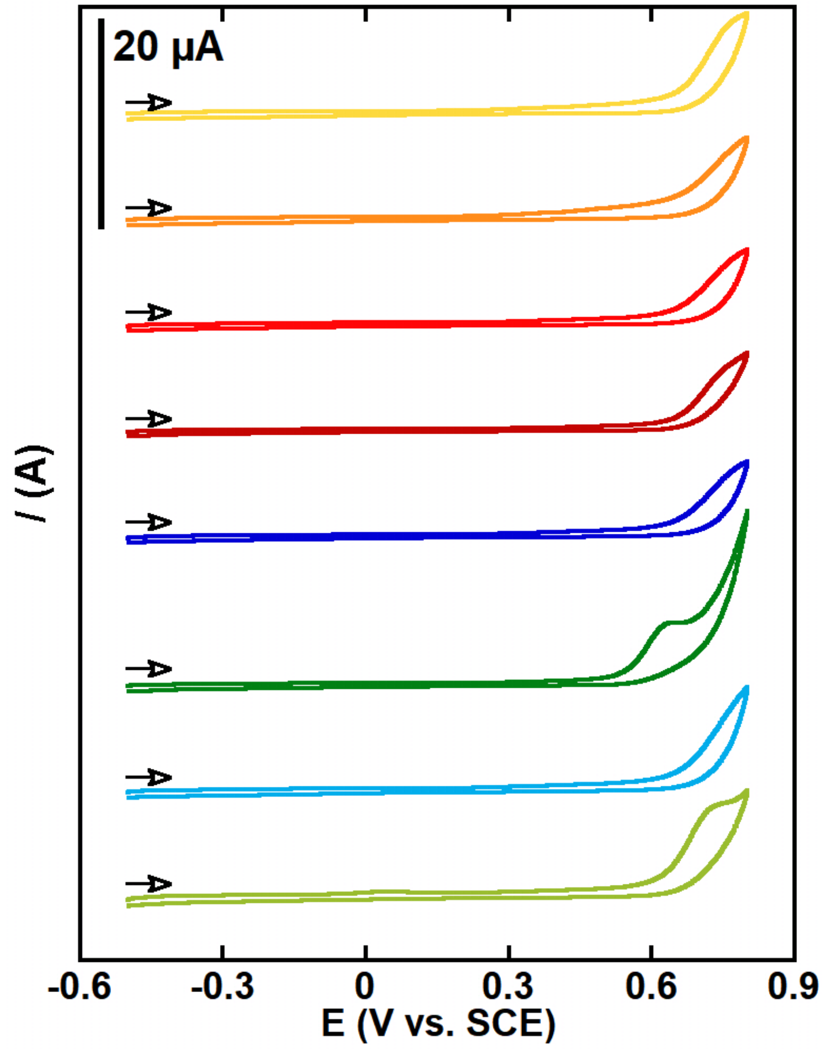

Figure S4._ Cyclic voltammograms of Cu(P), (GVHW (yellow curve), VGHW (orange curve), GGHW (red curve), DAHW (dark red curve), GHHW (dark blue curve), HGHW (light blue curve), WHHG (dark green curve), HWHG (light green curve)). The arrows indicate the scanning starting point going first in oxidation. [P] = 200 µM, $[Cu^{II}]$ = 180 µM, [HEPES] = 50 mM, pH 7.4, under argon. Scan rate = 100 $mV.s^{-1}$; WE = Glassy carbon, Ref = SCE, CE = Pt wire. No oxidation process can be seen before 0.6 V *vs*. SCE showing no electrochemical process. First scans are shown.

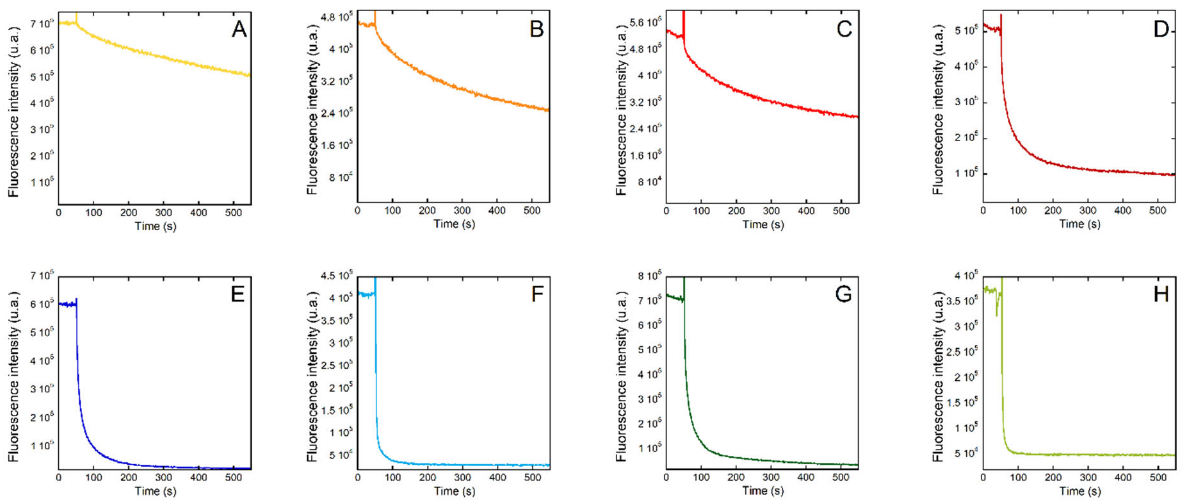

Figure S5. Kinetics of Cu^{II} removal from $Cu^{II}(A\beta)$ by GVHW (yellow curve), VGHW (orange curve), GGHW (red curve), DAHW (dark red curve), GHHW (dark blue curve), WHHG (dark green curve), HGHW (light blue curve), HWHG (light green curve) followed by fluorescence. The $Cu^{II}(A\beta)$ was added to a peptide solution at 50 s after the beginning of the kinetic. Experimental conditions: $[Cu^{II}(A\beta)]$ = [P] = 1 µM, [HEPES] = 100 mM, pH 7.4, T = 25 °C, λ_{ex} = 280 nm and λ_{em} = 350 nm.

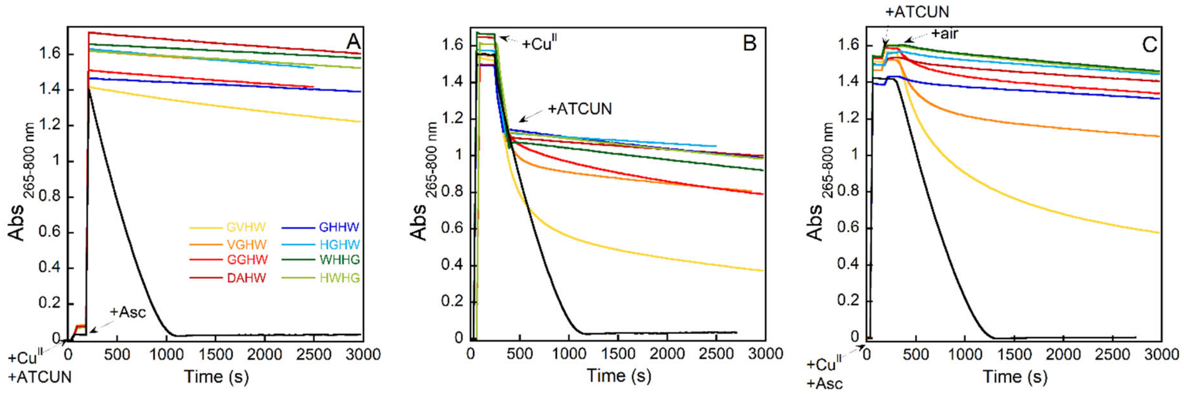

Figure S6. Kinetics of ascorbate consumption induced by copper followed by UV-visible spectroscopy at 265 nm starting from Cu^{II} (panel A), from $Cu^{II/I}$ (panel B) and from Cu^{I} (panel C). GVHW (yellow curve), VGHW (orange curve), GGHW (red curve), DAHW (dark red curve), GHHW (dark blue curve), HGHW (light blue curve), WHHG (dark green curve), HWHG (light green curve). [P] = 12 µM, [Cu^{II}] = 10 µM, [Asc] = 100 µM, [HEPES] = 100 mM, pH 7.4, T = 25 °C. The arrows indicate the time at which the different components are added into the cuvette.

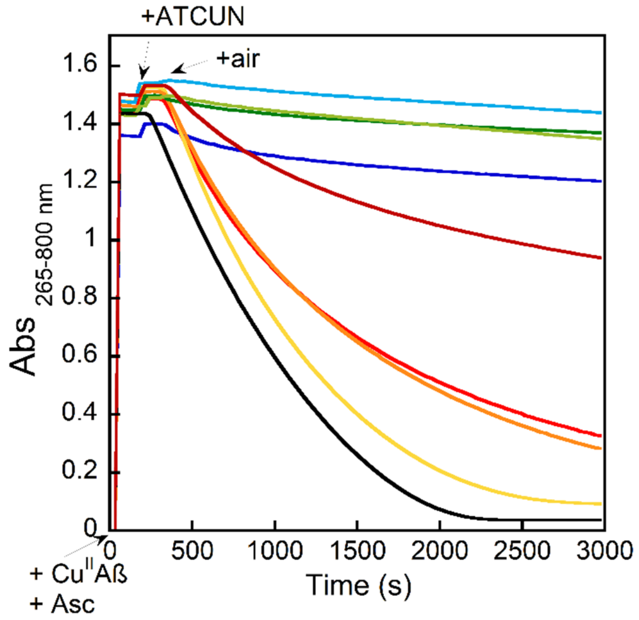

Figure S7. Kinetics of ascorbate consumption induced by Cu(Aβ) followed by UV-visible spectroscopy at 265 nm starting from Cu^{I}(Aβ) with 30 s of incubation with P (GVHW (yellow curve), VGHW (orange curve), GGHW (red curve), DAHW (dark red curve), GHHW (dark blue curve), HGHW (light blue curve), WHHG (dark green curve), HWHG (light green curve)). [P, Aβ] = 12 µM, [Cu^{II}] = 10 µM, [Asc] = 100 µM, [HEPES] = 100 mM, pH 7.4, T = 25 °C. The arrows indicate the time at which the different components are added into the cuvette.
